# Location and Level of Etk Expression in Neurons Are Associated with Varied Severity of Traumatic Brain Injury

**DOI:** 10.1371/journal.pone.0039226

**Published:** 2012-06-18

**Authors:** John Chung-Che Wu, Kai-Yun Chen, Yu-Wen Yu, Song-Wei Huang, Hsiu-Ming Shih, Wen-Ta Chiu, Yung-Hsiao Chiang, Chia-Yang Shiau

**Affiliations:** 1 Graduate Institute of Medical Sciences, National Defense Medical Center, Taipei, Taiwan, Republic of China; 2 Taitung Christian Hospital, Taitung, Taiwan, Republic of China; 3 Department of Neurosurgery, Taipei Medical University Hospital, Taipei, Taiwan, Republic of China; 4 Neural Regenerative Program, College of Medical Science and Technology, Taipei, Taiwan, Republic of China; 5 Translational Research Laboratory, Cancer Center, Taipei Medical University Hospital, Taipei, Taiwan, Republic of China; 6 Department of Surgery, College of Medicine, Taipei Medical University, Taipei, Taiwan, Republic of China; 7 Institute of Biomedical Sciences, Academia Sinica, Taipei, Taiwan, Republic of China; 8 Ministry of Health, Taipei, Taiwan, Republic of China; Max Planck Institute of Psychiatry, Germany

## Abstract

**Background:**

Much recent research effort in traumatic brain injury (TBI) has been devoted to the discovery of a reliable biomarker correlating with severity of injury. Currently, no consensus has been reached regarding a representative marker for traumatic brain injury. In this study, we explored the potential of epithelial/endothelial tyrosine kinase (Etk) as a novel marker for TBI.

**Methodology/Principal Findings:**

TBI was induced in Sprague Dawley (SD) rats by controlled cortical impact. Brain tissue samples were analyzed by Western blot, Q-PCR, and immunofluorescence staining using various markers including glial fibrillary acidic protein, and epithelial/endothelial tyrosine kinase (Etk). Results show increased Etk expression with increased number and severity of impacts. Expression increased 2.36 to 7-fold relative to trauma severity. Significant upregulation of Etk appeared at 1 hour after injury. The expression level of Etk was inversely correlated with distance from injury site. Etk and trauma/inflammation related markers increased post-TBI, while other tyrosine kinases did not.

**Conclusion/Significance:**

The observed correlation between Etk level and the number of impacts, the severity of impact, and the time course after impact, as well as its inverse correlation with distance away from injury site, support the potential of Etk as a possible indicator of trauma severity.

## Introduction

The discovery of a reliable biomarker correlating with severity of injury has been the focus of much of the recent research effort in traumatic brain injury (TBI). An ideal biomarker would exhibit a rapid change signifying sensitivity to disease, tissue specificity, and diagnostic value for the disease. For TBI, the biomarker would have added value if it could also be used to predict neurological condition and serve as a surrogate endpoint for evaluation of treatment. Currently, no consensus has been reached regarding a representative marker for traumatic brain injury [1]. Previous studies show significant post-TBI increases of S-100, glial fibrillary acidic protein (GFAP), neuron-specific enolase (NSE), neurofilament polypeptides, and tau. However, while these markers demonstrate potential as indicators of TBI [2], [3], [4], the study results showed a lack of correlation of these markers with clinical trauma severity [5], [6], [7], [8] and suggested limitations in the discriminative powerof some of these biomarkers alone [9].

**Figure 1:**
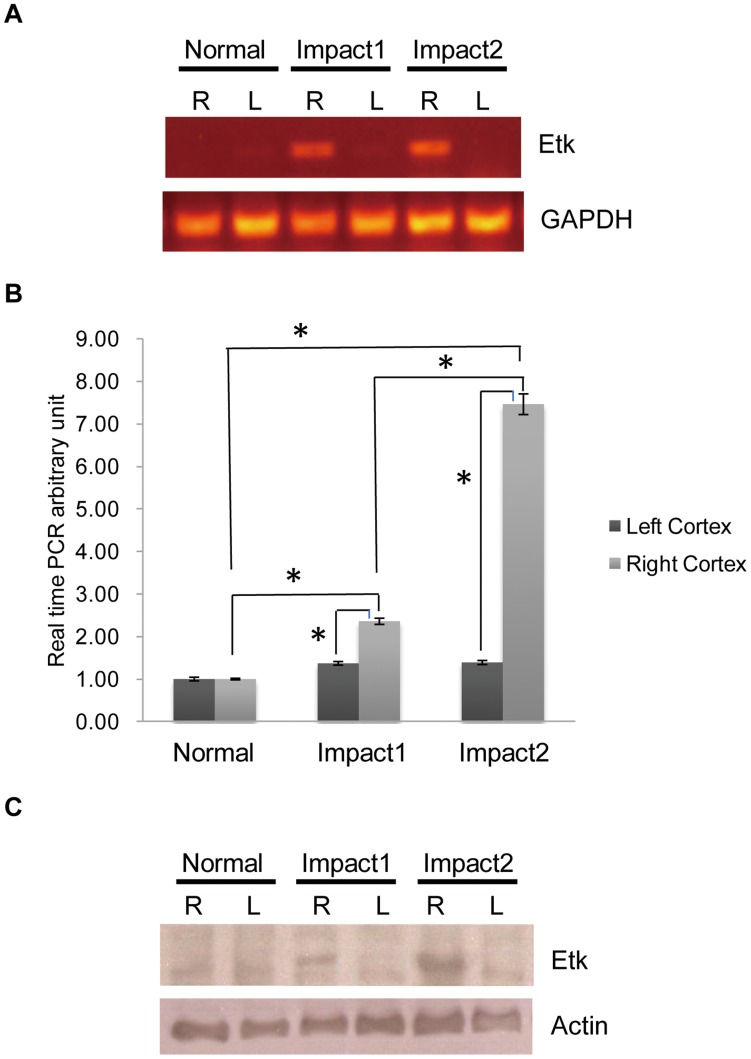
Etk is up-regulated after TBI. (A, B) Extracts of rat brains treated without impact (Normal), impacted once (impact1) or impacted twice (impact2) were subjected to real- time PCR analysis. Expression of GAPDH mRNA was used as internal control. The relative Etk level of the impact1 rat was used as the fold increase compared to left brain of normal rat. L  =  left brain. R  =  right brain. (C) Western blots were done with antibodies to Etk and actin (loading control).

Although the general pathophysiology of TBI is still unclear, recent research has revealed several possible mechanisms underlying TBI [1], [10]. While some studies suggest the disruption of the blood-brain barrier as a main cause of secondary injury [11], much of the literature focuses on mechanisms involving lipid peroxidation and the activation of calpain by the increase of intracellular calcium [12], [13], [14], [15]. Calpain is also known to activate Hsp70 and lysosomal release of cathepsin which results in axonal beading and diffuse axonal injury after TBI [16], [17].

Many therapeutic targets involving the cascade triggered by lipid peroxidation are currently under investigation for use in the treatment of TBI. Therapeutic strategies suggested include the reduction of mitochondrial free radical production [14], [15], [18], [19] and the scavenging of peroxynitrite-derived free radicals with tempol and melatonin [18], [20], [21]. The use of estrogen, progesterone, telmisartan and wogonin has also been suggested to limit damage secondary to TBI [22], [23], [24], [25]. Calpain activates many injury pathways with its proteolytic activity on myelin basic protein [26]and mediation of collapsin mediator proteins−1, −2, and −4 [27], and has been suggested as a potential target for TBI treatment [13], [14], [15], [27], [28], [29], [30], [31], [32].

Many peptides produced by proteolytic reactions caused by calpain activation, such as alpha-spectrin derivatives, have been considered as markers for TBI [33]. The elevation of calpain-derived alpha-spectrin among other markers in cerebrospinal fluid was observed at 24 hours after TBI with peak levels not reached until 48–96 hours [34]. As early detection of TBI severity is desirable [34], we sought to find other factors which may underly the initiation of injury.

**Figure 2:**
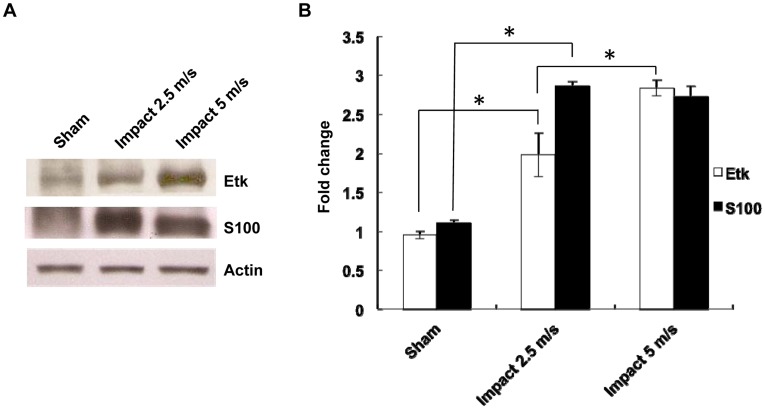
Correlation of Etk and S100 expression with severity of impact. (A, B) Western blots revealing expression of Etk was upregulated nearly 2-fold in rats receiving controlled cortical impact at 2.5 M/s and was further increased to 2.7-fold when impact speed was increased to 5M/s. S100 was increased to more than 2.5 fold after impact at these speeds.

A tyrosine kinase of interest is the tec kinase bone marrow tyrosine kinase gene in chromosome X (Bmx) which is also known as epithelial/endothelial tyrosine kinase (Etk) [35]. Most literature thus far regard Bmx/Etk as a modulator of apoptosis and cancer cell growth, and its cell-specific function has been characterized in various cancer cells [36]. Studies have also shown the Bmx/Etk-dependent pathway to be crucial in ischemic brain injury for the recruitment of inflammatory cells and angiogenesis at the site of injury [12], [37], [38]. Genetic profiling suggests that an increased expression of Bmx/Etk induces chronic inflammation and angiogenesis via cytokine-mediated recruitment of inflammatory cells [39]. In addition, Bmx/Etk was reported to regulate Toll-like receptor-induced IL-6 production, a cytokine closely related to traumatic brain injury [40], [41]. The possible role of Etk in post-traumatic neural injury and in the inflammation cascadeis the focus of this study.

**Figure 3:**
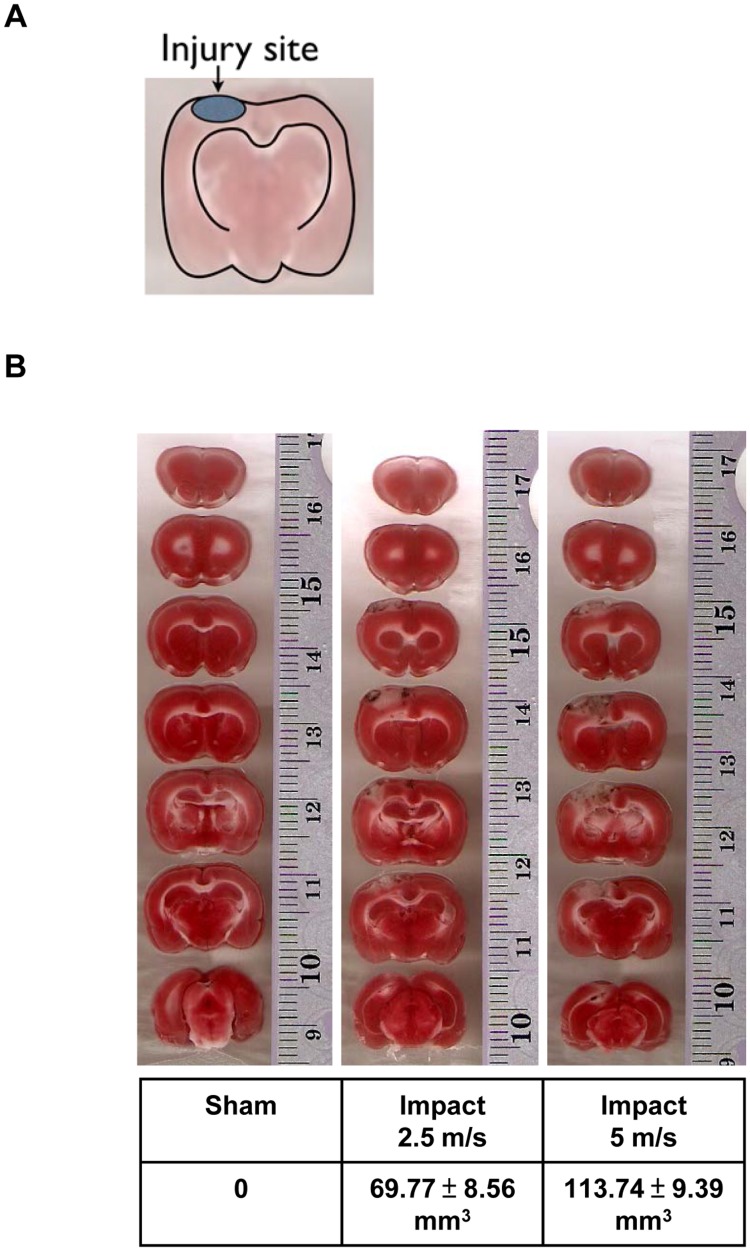
Triphenyltetrazolium chloride (TTC) staining of brain having received CCI. (A) Figure indicating location of site of induced trauma. (B) Size of injured cortex was also increased proportionally to the speed of impact using triphenyltetrazolium chloride (TTC) staining.

Some studies have implicated GFAP, S-100b, cleaved tau, and IL-6 as potential trauma markers [42], [43], [44], [45]; however, other reports have documented their limitations in clinical use [9], [46], [47]. An ideal biomarker should respond rapidly to the onset of disease and be diagnostic of the condition. Furthermore, an ideal biomarker should possess tissue specificity and be useful as a surrogate endpoint to address therapeutic efficacy [34]. Since our initial experiments with genetic profiling showed a moderate increase in Etk expression levels of 1.8 to 2.5 fold in rats with induced TBI compared to naïve rats (data not shown), we sought to clarify the correlation between level of Etk expression and degree of cranial trauma. In this study, we demonstrated the potential of Etk as a neurotrauma biomarker based on its expression correlating with the location and the degree of traumatic brain injury.

**Figure 4:**
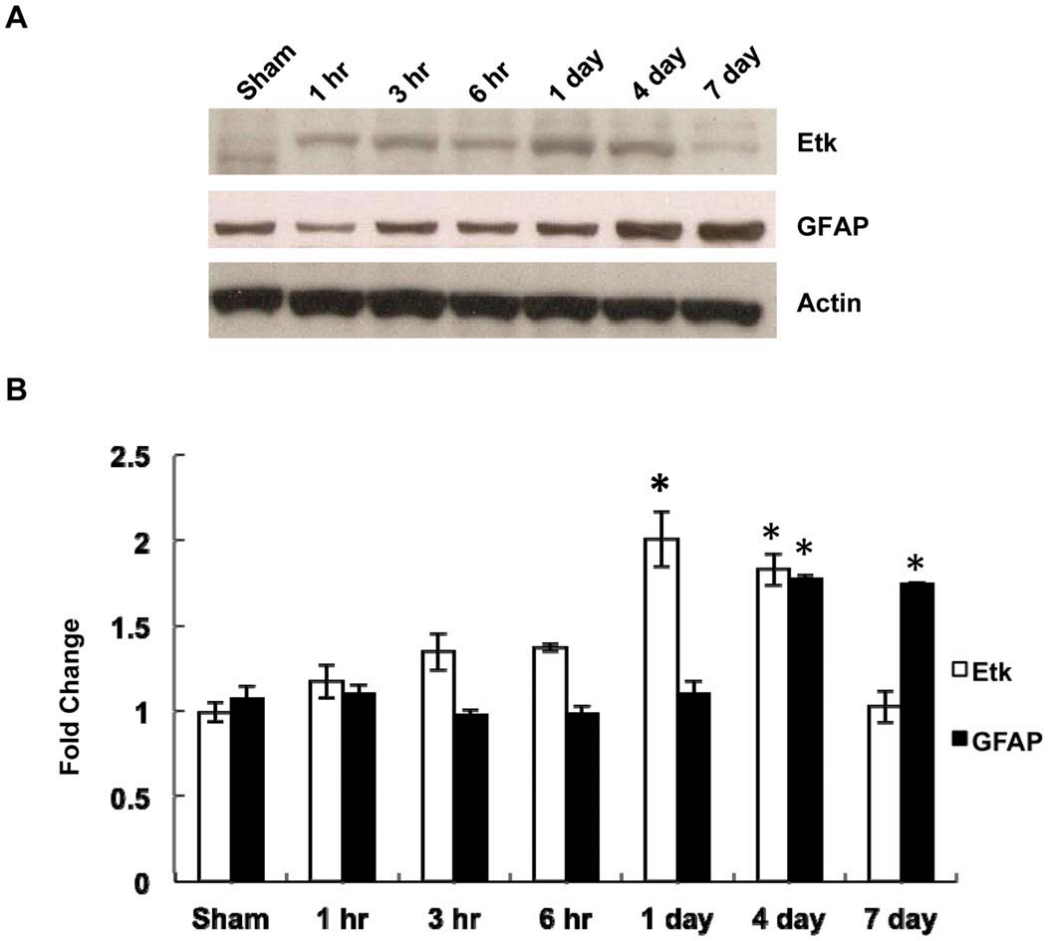
Time course of changes in Etk and GFAP levels after controlled cortical impact. (A) Western blot of Etk and GFAP expression at 1 hour, 3 hours, 6 hours, 4 days and 7 days after impact. (B) Densitometric analysis of Etk and GFAP western blot results revealing a significant increase at 3 hours after impact for Etk and at 4 days for GFAP. Values are expressed as mean±SD of three different experiments. *P<0.05 vs sham.

## Methods

### Western Blot

Brain tissue samples were lysed in RIPA buffer. 50–100 µg of cell lysates were resolved on 8% to 15% SDS/PAGE gel and transferred onto nitrocellulose membranes. Subsequently, blots were incubated with antibodies raised against the following proteins: anti-Bmx, anti-Stat3 (1∶1000, Transduction Laboratories), anti-Tec, anti-Btk, anti-Src, anti-FAK, anti- Bcl2, anti-LC3 (1∶1000, Cell Signaling) and Actin (1∶1000, Sigma). Donkey peroxidase-conjugated anti-rabbit or anti-mouse antibodies (1∶1000, Amersham Pharmacia Biotech) were used and binding was revealed by chemiluminescence (1∶1000, ECL; Amersham Pharmacia Biotech).

**Figure 5:**
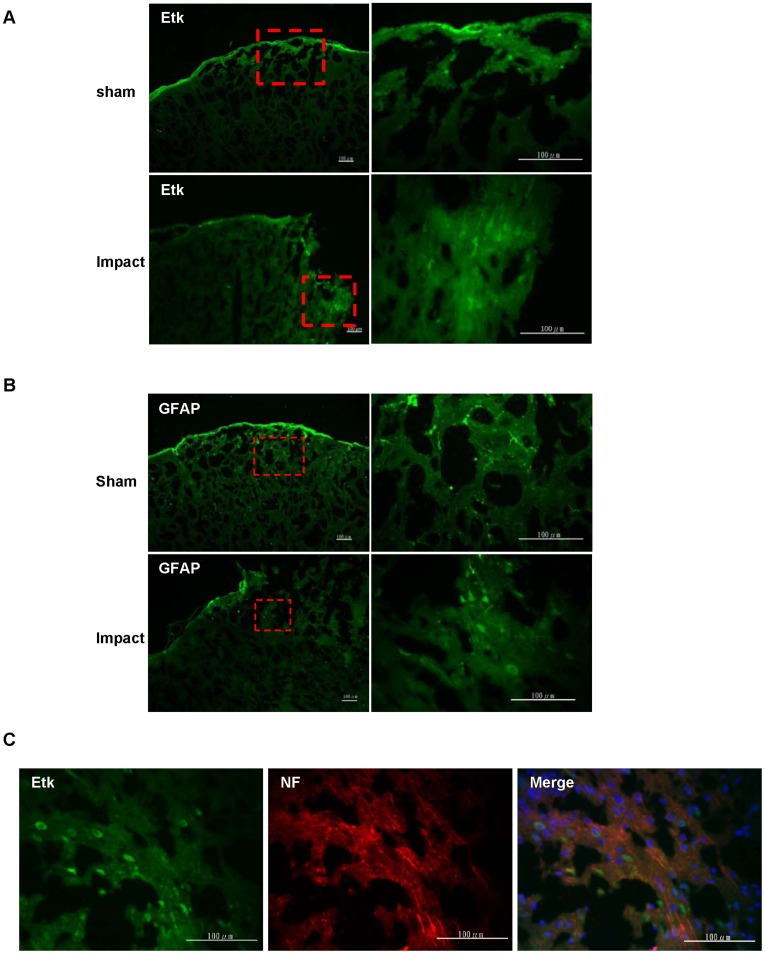
Immunofluorescence of Etk and GFAP at the injury site after controlled cortical impact. (A,B) Both Etk and GFAP was detectable near the injury site, and the intensity of the immunofluorescence signal for both proteins decreases as distance increases away from injury site. (C) Etk exhibits colocalization with neurofilament immunostaining.

### RT-PCR and Q-PCR

Total RNA was extracted from brain tissue by utilizing Trizol reagent (Invitrogen). Prior to RT-PCR, 1 µg of RNA was initially treated with DNase I (Ambion Inc., Austin, TX) to degrade genomic DNA. Thereafter, 50 ng of treated RNA was used for each one-step RT-PCR reaction (QIAGEN OneStep RT-PCR Kit, Valencia, CA). Gene expression was quantified by QRT-PCR using SYBR Green dye. All QRT-PCR reactions were performed on a 7900 HT ABI platform (Applied Biosystems, Foster City, CA) as previously described [48]. The sequences of primers were as follows: GAPDH forward 5′-GCACCGTCAAGGCTGAGAAC-3′ and reverse 5′-ATGGTGGTGAAGACGCCA-3′. GAPDH was used to normalize the expression levels in the quantitative analyses. The forward primer for mEtk was 5'-CACACCACCTCAAAGATTTCATGG-3' and the reverse primer was 5'-CATACTGCCCCTTCCACTTGC-3'.

**Figure 6:**
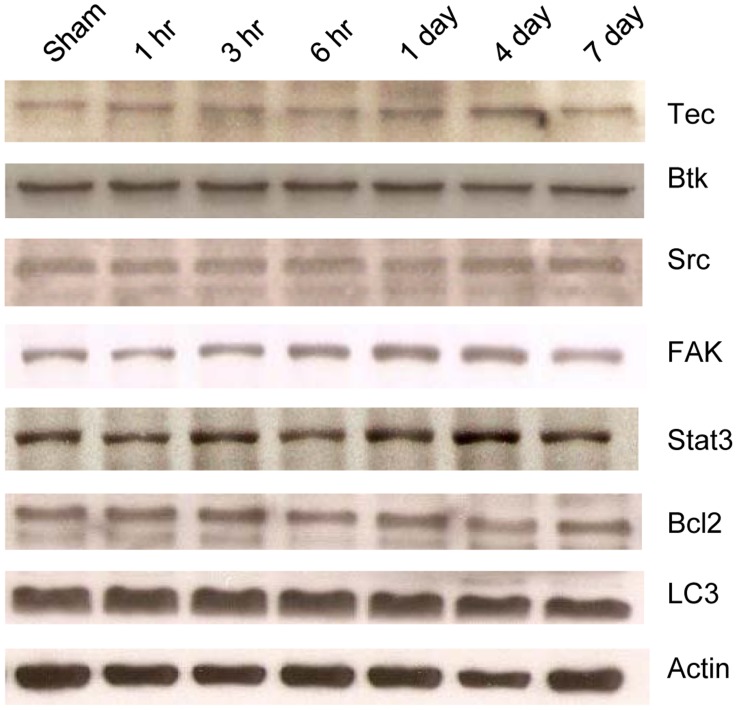
Western blot analysis for other proteins at various times after impact. Other tyrosine kinases (Tec, Btk, Src, FAK)and signal transduction proteins (Stat3, Bcl2, LC3 ) were analyzed after impact, Etk was uniquely upregulated after injury.

### Controlled-Cortical Impact

Animals were sedated prior to impact and treated according to a Taipei Medical University Laboratory Animal protocol. Animal studies were approved by the Institutional Animal Care and Use Committee (IACUC) of National Defense Medical Center Laboratory Animal Center (NDMCLAC). Adult male Sprague-Dawley rats (weight 280–300 g) were used for this study. The surgical procedures were modified from the Lin CM et al. (2009) method. Under chloral hydrate (40 mg/kg, intraperitoneal, i.p., injection, Kanto Chemical Co., Inc.) anesthetized, SD rats were placed in a stereotactic frame. A craniotomy of 5 mm diameter was performed at the right parietal cortex between bregma and lambda, and 1 mm lateral from the midline. TBI was made by a controlled cortical impact (CCI) device at a velocity of 2.5 or 5 m/sec with 1 mm depth. Body temperature was maintained at 37°C ±1°C with a heating pad. Rats were sacrificed at appropriate times for analysis, and assignments of groups were blinded to observers. For TTC staining, animals were killed at 24 hours after impact, the brains were removed and a series of 2-mm coronal slices were obtained and stained in 2% triphenyltetrazolium chloride (Sigma) in 0.9% saline, then fixed in 4% paraformaldehyde. The injury area, which was not stained, was measured using a digital scanner as previously described [49].

### Immunofluorescence

Twenty-four hours after TBI, rats were perfused through the ascending aorta with 100 mL of cold normal saline followed by 100 mL of 4% paraformaldehyde (PFA) in PBS. Brains were removed and post-fixed in the same fixative for 3 days followed by 30% sucrose for 1 week. Sections were cut at a thickness of 12 microns in a freezing microtome and stored at −20°C. For immunostaining, tissue sections were fixed with 4% PFA for 10 minutes. After several washes in PBS, the sections were incubated with blocking buffer containing 0.3% Triton X−100 and 4% bovine serum albumin for 1 hour at room temperature, and were then stained with the desired primary antibody reconstituted in PBS, 2% goat serum at 4°C for 14–16 hours. Dilutions of the anti-Etk (Cell Signaling), anti-neurofilament M (NF), anti-GFAP (Transduction Laboratories) antibodies were 1∶100. After three rinses in PBS, sections were incubated with goat anti-rabbit IgG FITC conjugate (1∶100 Jackson Immunoresearch) and goat anti-mouse IgG Rhodamine conjugate (1∶100; Jackson Immunoresearch) for 1 hour at room temperature. 1mg/mL DAPI was added to the mixture during the last 15 minutes. After several washes in PBS, sections were mounted with Crystal Mount (Sigma) and analyzed using a Leica microscope, a SROT RTTM CCD camera (Diagnostic Instruments) or laser-scanning confocal microscope (Bio-Rad, MRC-1000).

### Statistics

Data are presented as mean ± SD. Oneway ANOVA and post-hoc Newman-Keuls tests were used for statistical comparison. A statistically significant difference was defined at p<0.05.

## Results

### Impact Increased Etk Expression Compared to the Contralateral Hemisphere

PCR product (Figure 1A), real-time PCR analysis(Figure 1B), and Western blot analysis(Figure 1C) demonstrated that Etk expression is increased post-impact injury when compared to the normal cortex. Upregulated Etk expression levels were observed after trauma by both PCR and Western blot analyses (Figure 1A, C). The expression of Etk increased in the single impact groups by 2.36-fold (Figure 1B). Expression increased up to 7fold when impact was performed twice. (Figure 1B, *p<0.05, One-Way ANOVA, posthoc Newman-Keuls test).

### Expression of Etk is Related to Trauma Severity as shown by Western Blot Analysis

Since an increase in Etk level was observed upon impact with a marked further increase observed upon second impact, the correlation of the expression of Etk with trauma severity was examined. Differing degrees of cortical injury were induced using the CCI model with 2.5m/s and 5m/s speed settings. Although S100 increased after impact, the level of increase did not vary among the different trauma severity groups. Conversely, the increase in the level of Etk upregulation upon impact showed statistically significant differences between groups with varied trauma severity (Figure 2A, 2B, *p<0.05, One-Way ANOVA, posthoc Newman-Keuls test). Western blot analysis of Etk correlated with the degree of injury severity revealed by TTC staining. (Figure 3).

### Etk and GFAP Increase with Respect to Time after TBI shown by Western Blot Analysis

The expression of Etk and GFAP increased with respect to time after injury. GFAP responded at a later stage and lasted for up to 7 days. In contrast, Etk upregulation appeared significant at 1 hour post-injury and continued to increase until 4 days after injury (Figure 4A, 4B, *p<0.05, One-Way ANOVA, post hoc Newman-Keuls test).

### Location of Etk and GFAP with Respect to Injury Site shown by Immunofluorescence Analysis

Etk and GFAP were localized in the impacted hemisphere receiving mild CCI evidenced by increased fluorescence signals for Etk and GFAP near the injury site. Levels of expression decreased as distance from the injury site increased (Figure 5A, 5B). Etk also exhibited colocalization with neurofilament immunostaining (Figure 5C), suggesting the increase in Etk occurs in neurons. These findings suggest the increase in Etk level arises from direct injury to the neurons at the injury site.

### Only Etk and Trauma/inflammation Related Markers, but not Other Tyrosine Kinases, Increased after TBI

Western blot analyses at various times after TBI in rats revealed increased levels of only trauma related markers and Etk. Other tyrosine kinases and signal transduction proteins such as Tec, Btk, Src, FAK, Stat 3, Bcl2, LC3 appeared unchanged after trauma (Figure 6). The increase in Etk, but not other proteins, suggests that induction of Etk is specific for traumatic brain injury.

## Discussion

### Etk may be an Indicator for Trauma Severity

Our results support the correlation of Etk upregulation with trauma severity in rats. Based on the increase in Etk expression in the injured cortex post-impact demonstrated by Western blot, PCR, and RT-PCR, we postulate that Etk is associated with traumatic brain injury. The correlation between the levels of Etk expression with severity of injury was demonstrated by using different degrees of controlled cortical impact. Furthermore, the level of Etk increased as early as 1 hour after injury and a gradual increase continued for 3 days or more. These increases in Etk expression were further demonstrated by immunostaining and correlated inversely with distance from the injury site. Taken together, the increase in Etk observed with the increased number of impacts, the severity of impact, and its time course after impact as well as its inverse correlation with distance away from injury support the possible role of Etk as a potential indicator for traumatic neural injury severity.

### Comparison to Other Markers

S100 and GFAP are two of the more accepted markers for neural injury. Although both Etk and S100 increased after trauma, a difference in degree of increase with respect to injury severity was not observed for S100, yet was clearly demonstrated in the expression of Etk. Furthermore, although both Etk and GFAP expression demonstrated a timedependent increase after trauma, the increase in Etk expression levelwas statistically significant at 3 hours after trauma. In contrast, the level of expression for GFAP was not significantly different at 1 day post-trauma but increased nearly 2- fold at 4 days post-trauma. With immunostaining of GFAP and Etk, the difference between the two was equally apparent at the site of injury, and both exhibited decreasing expression at distances further away from the injury site.

### Etk is Uniquely Upregulated by TBI and may be a Potential Neurotrauma Biomarker

The upregulation of Etk is both temporally and spatially correlated with injury. The upregulation responded more rapidly to injury compared to GFAP.Similar post-trauma upregulation was not observed with other tyrosine kinases of the same class or with several other major signal transduction proteins in our study. Thus, Etk appears to be uniquely upregulated after trauma and may be a marker indicating trauma severity.

There are still several obstacles to be overcome before Etk can be developed into a clinical biomarker for TBI severity. As current detection of Etk in serum still has unresolved problems, we are currently investigating the possibility of detecting substrates of Etk as an alternative. In addition, while the expression of Etk is present in other tissues such as bone marrow, and may be elevated even in the absence of neuronal injury, its unique increase after TBI may still provide pertinent information when used in combination with other nerual or non-neural trauma markers.

In conclusion, unique Etk upregulation with respect to traumatic neural injury severity suggests a possible role of Etk as a neurotrauma marker.Its feasibility as an indicator for neurotrauma severity in human clinical settings warrants further clinical investigation.

## References

[1] Andriessen TM, Jacobs B, Vos PE (2010). Clinical characteristics and pathophysiological mechanisms of focal and diffuse traumatic brain injury.. Journal of cellular and molecular medicine.

[2] de KruijkJR, Leffers P, Menheere PP, Meerhoff S, Twijnstra A (2001). S-100B and neuron-specific enolase in serum of mild traumatic brain injury patients. A comparison with health controls.. Acta Neurol Scand.

[3] Ingebrigtsen T, Romner B (2003). Biochemical serum markers for brain damage: a short review with emphasis on clinical utility in mild head injury.. Restor Neurol Neurosci.

[4] Lo TY, Jones PA, Minns RA (2009). Pediatric brain trauma outcome prediction using paired serum levels of inflammatory mediators and brain-specific proteins.. J Neurotrauma.

[5] Woertgen C, Rothoerl RD, Wiesmann M, Missler U, Brawanski A (2002). Glial and neuronal serum markers after controlled cortical impact injury in the rat.. Acta Neurochir.

[6] Rothoerl RD, Brawanski A, Woertgen C (2000). S-100B protein serum levels after controlled cortical impact injury in the rat.. Acta Neurochir (Wien).

[7] Geyer C, Ulrich A, Grafe G, Stach B, Till H (2009). Diagnostic value of S100B and neuron-specific enolase in mild pediatric traumatic brain injury.. J Neurosurg Pediatr.

[8] Pelinka LE, Hertz H, Mauritz W, Harada N, Jafarmadar M (2005). Nonspecific increase of systemic neuron-specific enolase after trauma: clinical and experimental findings.. Shock.

[9] Topolovec-Vranic J, Pollmann-Mudryj MA, Ouchterlony D, Klein D, Spence J (2011). The value of serum biomarkers in prediction models of outcome after mild traumatic brain injury.. The Journal of trauma.

[10] Risdall JE, Menon DK (2011). Traumatic brain injury.. Philosophical transactions of the Royal Society of London Series B, Biological sciences.

[11] Zhu D, Wang Y, Singh I, Bell RD, Deane R (2010). Protein S controls hypoxic/ischemic blood-brain barrier disruption through the TAM receptor Tyro3 and sphingosine 1-phosphate receptor.. Blood.

[12] Chen KY, Wu CC, Chang CF, Chen YH, Chiu WT (2011). Suppression of Etk/Bmx Protects against Ischemic Brain Injury.. Cell transplantation.

[13] Xiong Y, Hall ED (2009). Pharmacological evidence for a role of peroxynitrite in the pathophysiology of spinal cord injury.. Experimental neurology.

[14] Deng Y, Thompson BM, Gao X, Hall ED (2007). Temporal relationship of peroxynitrite-induced oxidative damage, calpain-mediated cytoskeletal degradation and neurodegeneration after traumatic brain injury.. Experimental neurology.

[15] Xiong Y, Rabchevsky AG, Hall ED (2007). Role of peroxynitrite in secondary oxidative damage after spinal cord injury.. Journal of neurochemistry.

[16] Yamashima T, Oikawa S (2009). The role of lysosomal rupture in neuronal death.. Progress in neurobiology.

[17] Kilinc D, Gallo G, Barbee KA (2009). Mechanical membrane injury induces axonal beading through localized activation of calpain.. Experimental neurology.

[18] Deng-Bryant Y, Singh IN, Carrico KM, Hall ED (2008). Neuroprotective effects of tempol, a catalytic scavenger of peroxynitrite-derived free radicals, in a mouse traumatic brain injury model.. Journal of cerebral blood flow and metabolism : official journal of the International Society of Cerebral Blood Flow and Metabolism.

[19] Zhou P, Qian L, D'Aurelio M, Cho S, Wang G (2012). Prohibitin reduces mitochondrial free radical production and protects brain cells from different injury modalities.. The Journal of neuroscience : the official journal of the Society for Neuroscience.

[20] Das A, Belagodu A, Reiter RJ, Ray SK, Banik NL (2008). Cytoprotective effects of melatonin on C6 astroglial cells exposed to glutamate excitotoxicity and oxidative stress.. Journal of pineal research.

[21] Samantaray S, Sribnick EA, Das A, Knaryan VH, Matzelle DD (2008). Melatonin attenuates calpain upregulation, axonal damage and neuronal death in spinal cord injury in rats.. Journal of pineal research.

[22] Sribnick EA, Matzelle DD, Ray SK, Banik NL (2006). Estrogen treatment of spinal cord injury attenuates calpain activation and apoptosis.. Journal of neuroscience research.

[23] Cekic M, Johnson SJ, Bhatt VH, Stein DG (2012). Progesterone treatment alters neurotrophin/proneurotrophin balance and receptor expression in rats with traumatic brain injury.. Restorative neurology and neuroscience.

[24] Kasahara Y, Taguchi A, Uno H, Nakano A, Nakagomi T (2010). Telmisartan suppresses cerebral injury in a murine model of transient focal ischemia.. Brain research.

[25] Chen CC, Hung TH, Wang YH, Lin CW, Wang PY (2012). Wogonin Improves Histological and Functional Outcomes, and Reduces Activation of TLR4/NF-kappaB Signaling after Experimental Traumatic Brain Injury.. PloS one.

[26] Ottens AK, Golden EC, Bustamante L, Hayes RL, Denslow ND (2008). Proteolysis of multiple myelin basic protein isoforms after neurotrauma: characterization by mass spectrometry.. Journal of neurochemistry.

[27] Zhang Z, Ottens AK, Sadasivan S, Kobeissy FH, Fang T (2007). Calpain-mediated collapsin response mediator protein−1, −2, and −4 proteolysis after neurotoxic and traumatic brain injury.. Journal of neurotrauma.

[28] Mustafa AG, Wang JA, Carrico KM, Hall ED (2011). Pharmacological inhibition of lipid peroxidation attenuates calpain-mediated cytoskeletal degradation after traumatic brain injury.. Journal of neurochemistry.

[29] Thompson SN, Carrico KM, Mustafa AG, Bains M, Hall ED (2010). A pharmacological analysis of the neuroprotective efficacy of the brain- and cell-permeable calpain inhibitor MDL-28170 in the mouse controlled cortical impact traumatic brain injury model.. Journal of neurotrauma.

[30] Saatman KE, Creed J, Raghupathi R (2010). Calpain as a therapeutic target in traumatic brain injury.. Neurotherapeutics : the journal of the American Society for Experimental NeuroTherapeutics.

[31] Yu CG, Joshi A, Geddes JW (2008). Intraspinal MDL28170 microinjection improves functional and pathological outcome following spinal cord injury.. Journal of neurotrauma.

[32] Ai J, Liu E, Wang J, Chen Y, Yu J (2007). Calpain inhibitor MDL-28170 reduces the functional and structural deterioration of corpus callosum following fluid percussion injury.. Journal of neurotrauma.

[33] Pineda JA, Lewis SB, Valadka AB, Papa L, Hannay HJ (2007). Clinical significance of alphaII-spectrin breakdown products in cerebrospinal fluid after severe traumatic brain injury.. Journal of neurotrauma.

[34] Siman R, Toraskar N, Dang A, McNeil E, McGarvey M (2009). A panel of neuron-enriched proteins as markers for traumatic brain injury in humans.. Journal of neurotrauma.

[35] Robinson D, He F, Pretlow T, Kung HJ (1996). A tyrosine kinase profile of prostate carcinoma.. Proc Natl Acad Sci U S A.

[36] Chen KY, Huang LM, Kung HJ, Ann DK, Shih HM (2004). The role of tyrosine kinase Etk/Bmx in EGF-induced apoptosis of MDA-MB-468 breast cancer cells.. Oncogene.

[37] He Y, Luo Y, Tang S, Rajantie I, Salven P (2006). Critical function of Bmx/Etk in ischemia-mediated arteriogenesis and angiogenesis.. The Journal of clinical investigation.

[38] Jasielska M, Semkova I, Shi X, Schmidt K, Karagiannis D (2010). Differential role of tumor necrosis factor (TNF)-alpha receptors in the development of choroidal neovascularization.. Investigative ophthalmology & visual science.

[39] Paavonen K, Ekman N, Wirzenius M, Rajantie I, Poutanen M (2004). Bmx tyrosine kinase transgene induces skin hyperplasia, inflammatory angiogenesis, and accelerated wound healing.. Mol Biol Cell.

[40] Helmy A, Carpenter KL, Skepper JN, Kirkpatrick PJ, Pickard JD (2009). Microdialysis of cytokines: methodological considerations, scanning electron microscopy, and determination of relative recovery.. J Neurotrauma.

[41] Palmer CD, Mutch BE, Workman S, McDaid JP, Horwood NJ (2008). Bmx tyrosine kinase regulates TLR4-induced IL-6 production in human macrophages independently of p38 MAPK and NFkapp}B activity.. Blood.

[42] Kleindienst A, Hesse F, Bullock MR, Buchfelder M (2007). The neurotrophic protein S100B: value as a marker of brain damage and possible therapeutic implications.. Prog Brain Res.

[43] Pelinka LE, Toegel E, Mauritz W, Redl H (2003). Serum S 100 B: a marker of brain damage in traumatic brain injury with and without multiple trauma.. Shock.

[44] Gabbita SP, Scheff SW, Menard RM, Roberts K, Fugaccia I (2005). Cleaved-tau: a biomarker of neuronal damage after traumatic brain injury.. J Neurotrauma.

[45] Hergenroeder GW, Moore AN, McCoy JP, Jr., Samsel L, Ward NH, 3rd, et al (2010). Serum IL-6: a candidate biomarker for intracranial pressure elevation following isolated traumatic brain injury.. J Neuroinflammation.

[46] Honda M, Tsuruta R, Kaneko T, Kasaoka S, Yagi T (2010). Serum glial fibrillary acidic protein is a highly specific biomarker for traumatic brain injury in humans compared with S-100B and neuron-specific enolase.. J Trauma.

[47] Gyorgy AB, Ling GS, Wingo DL, Walker J, Tong LC (2011). Time-dependent changes in serum biomarker levels after blast traumatic brain injury.. J Neurotrauma.

[48] Tran ND, Kim S, Vincent HK, Rodriguez A, Hinton DR (2010). Aquaporin-1-mediated cerebral edema following traumatic brain injury: effects of acidosis and corticosteroid administration.. Journal of neurosurgery.

[49] Wang Y, Lin SZ, Chiou AL, Williams LR, Hoffer BJ (1997). Glial cell line-derived neurotrophic factor protects against ischemia-induced injury in the cerebral cortex.. The Journal of neuroscience : the official journal of the Society for Neuroscience.

